# Saving Hearts in Rural West Texas: The Impact of Critical Care Access on Mortality Rates

**DOI:** 10.7759/cureus.55253

**Published:** 2024-02-29

**Authors:** Rami Al-Ayyubi, Laura Gonzales-Reyes, Andres Mata, Divya Parepalli, Muhammad Qudrat Ullah, Aimen Dar, Raghavendra Sanivarapu

**Affiliations:** 1 Internal Medicine, Texas Tech University Health Sciences Center Permian Basin, Odessa, USA; 2 Cardiovascular Medicine, Texas Tech University Health Sciences Center Lubbock, Lubbock, USA; 3 Pulmonary and Critical Care Medicine, Texas Tech University Health Sciences Center Permian Basin, Midland, USA

**Keywords:** west texas, rural hospitals, age-adjusted mortality rate, heart failure hospitalization, critical access hospital

## Abstract

Background

The Critical Access Hospital (CAH) designation program was created in 1997 by the US Congress to reduce the financial vulnerability of rural hospitals and improve access to healthcare by keeping fundamental services in rural communities.

Methods

This is a retrospective observational study. Information on CAHs in West Texas in rural counties was extrapolated from the Flex Monitoring Team between 2010 and 2020. The study population included adults aged ≥25 years with a known heart failure (HF) diagnosis who were identified using ICD-10 codes. Mortality rates were obtained from the CDC Wide-ranging ONline Data for Epidemiologic Research (WONDER) database. The HF population was categorized by age, sex, and ethnicity. Mortality differences among these groups were analyzed using a two-sample t-test. The significance level was considered to be p < 0.05.

Results

The total study population analyzed was 1,348,001. A statistically significant difference in age-adjusted mortality rate (AAMR) was observed between the study and control groups, with a value of 3.200 (95% CI: 3.1910-3.2090, p < 0.0001) in favor of a lower mortality rate in rural counties with CAHs. When comparing gender-related differences, males and females had lower AAMRs in rural counties with CAHs. Among each gender, statistically significant differences were noted between males (95% CI: 2.181-2.218, p < 0.001) and females (95% CI: 3.382-3.417, p < 0.001). When examining the data by ethnicity, the most significant difference in mortality rate was observed within the Hispanic population, 6.400 (95% CI: 6.3770-6.4230, p < 0.0001). When adjusted to age, the crude mortality rate was calculated, which favored CAH admission in the younger population (10.200 (95% CI: 10.1625-10.2375, p < 0.001) and 11.500 (95% CI: 11.4168-11.5832, p < 0.001) in the 55-64 and 65-74 age groups, respectively).

Conclusion

The data clearly showed that West Texas rural county hospitals that received CAH designation performed better in terms of mortality rates in the HF population compared to non-CAH.

## Introduction

The Critical Access Hospital (CAH) designation program was created in 1997 in response to rural hospital closures [1]. The US Congress started it to reduce the financial vulnerability of rural hospitals and improve access to healthcare by keeping fundamental services in rural communities. Such designation is given by the Centers for Medicare & Medicaid Services (CMS) to those hospitals that meet the following criteria: have 25 or fewer acute care inpatient beds; are located more than 35 miles from another hospital; maintain an annual average length of stay of 96 hours or less for critical patients; and provide 24/7 emergency care services [2].

Even though the CAH program has been around for almost 25 years, there is limited data to evaluate outcomes. The available data suggest that the financial efforts made by the CMS to expand CAH and support rural communities did not translate into higher quality of care in terms of mortality and readmission rates [3]. Data has shown that in some cases, performance measures and mortality rates are worse in CAH [4]. In a study by Joynt et al., CAH had higher mortality rates than non-CAH (13.3% vs. 11.4%; difference: 1.8%), and when comparing CAH with other small, rural hospitals, similar patterns were observed [4].

The non-reassuring outcomes raise significant concerns regarding highly prevalent diseases such as heart failure (HF). Unfortunately, HF remains one of the biggest healthcare concerns in the West Texas region. According to the CDC data, only five West Texas counties showed <200 per 100,000 death rates from HF from 2018 to 2020. Moreover, the HF hospitalization rate remains at a nationwide high (16.4 in Texas compared to 16.3 nationwide per 100,000 Medicare beneficiaries) [5].

There are 82 CAH facilities in Texas. Specifically, in rural West Texas, only 13 out of 25 county medical centers possess the CAH designation [6]. There needs to be a current data analysis regarding the quality of care patients with HF receive in West Texas. Thus, the investigators of this study aimed to compare the mortality rates of HF between the CAH and non-CAH in rural West Texas.

## Materials and methods

This retrospective observational study intends to analyze the difference in mortality rates in patients with HF in 25 West Texas rural counties admitted to local hospitals between 2010 and 2020.

The 2013 urbanization classification of non-metropolitan (rural) counties includes micropolitan counties (counties with an urban cluster population of 10,000-49,000 residents) and noncore counties (counties that cannot be classified as metropolitan or micropolitan). Twenty-five rural counties in West Texas were identified using the 2013 urbanization classification available via the CDC Wide-ranging ONline Data for Epidemiologic Research (WONDER) and were divided into two study groups. The study group was chosen to be HF patients admitted to 13 rural CAHs, while the control group was HF patients admitted to 12 rural non-CAHs. The information on CAH in West Texas was extrapolated from the Flex Monitoring Team Project [6].

The age cutoff was ≥25 years of age. Only patients with HF were chosen for this study using ICD codes I11.0 (hypertensive heart disease with (congestive) HF), I13.0 (hypertensive heart and renal disease with (congestive) HF), I13.2 (hypertensive heart and renal disease with both (congestive) HF and renal failure), I50.0 (congestive HF), I50.1 (left ventricular failure), and I50.9 (HF, unspecified), which were obtained from open-source data [5]. Mortality rates for our study population were obtained from the CDC WONDER database. The HF population was categorized by age group, sex, race, and ethnicity. To determine whether age-adjusted and crude mortality rates (CMRs) differed between the two study populations, rural counties with CAH and rural counties without were analyzed using Microsoft Excel (Microsoft Corporation, Redmond, Washington, United States) and a two-sample t-test. Online calculators were utilized for the application of a two-sample t-test. The significance level was considered to be p < 0.05.

## Results

**Figure 1:**
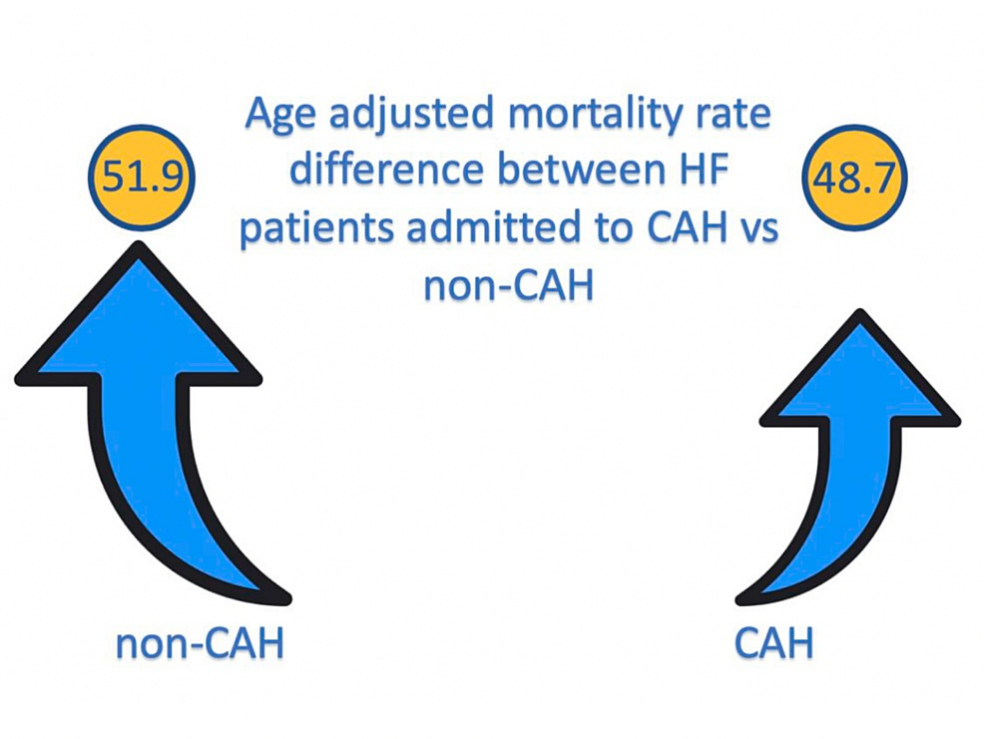
This shows a statistically significant difference in AAMR between the study and control groups, with a value of 3.200 (95% CI: 3.1910-3.2090, p < 0.0001) in favor of a lower mortality rate in rural counties with CAHs. AAMR, age-adjusted mortality rate; CAH, Critical Access Hospital; HF, heart failure

The study included a population of 1,348,001 individuals 25 years of age and older. Of the study population, 54.11% were male and 45.89% were female. A total of 735 deaths attributed to HF occurred between 2010 and 2020 in 25 rural West Texas county hospitals. Death rates were calculated per 100,000 persons. The age-adjusted mortality rate (AAMR) for the study population admitted to CAH was 48.7 (95% CI: 43.8-53.6, SE: 2.5). In contrast, the control group had a higher AAMR of 51.9 (95% CI: 46.5-57.3, SE: 2.8). A statistically significant difference in AAMR was observed between the two groups, with a value of 3.200 (95% CI: 3.1910-3.2090, p < 0.0001) in favor of a lower mortality rate in CAH (Figure 1).

**Figure 2:**
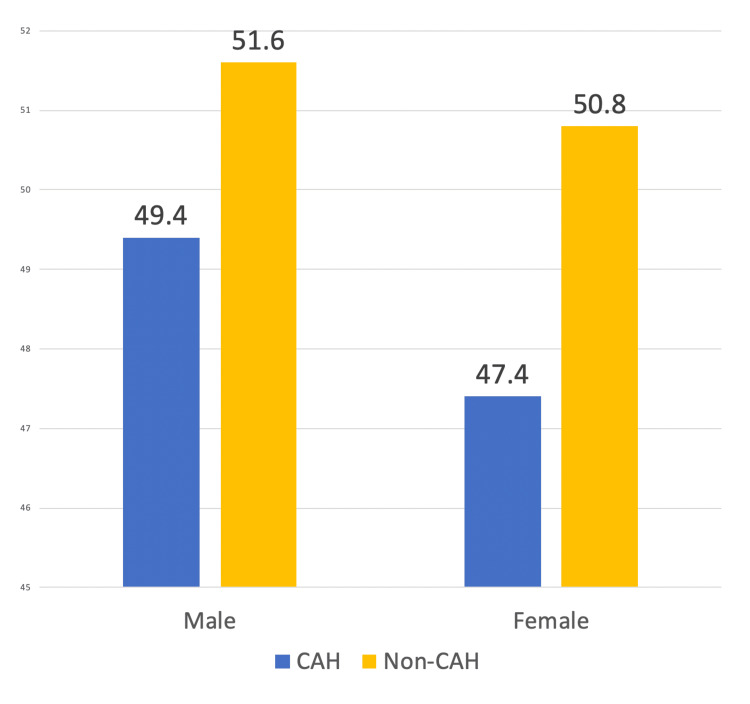
The bar graph shows gender-related differences in males and females. Both had lower AAMRs in rural counties with CAHs. Among each gender, statistically significant differences were noted between males (95% CI: 2.181-2.218, p < 0.001) and females (95% CI: 3.382-3.417, p < 0.001). AAMR, age-adjusted mortality rate; CAH, Critical Access Hospital

Additionally, our study observed almost 3.5 times higher AAMR among women with HF admitted to non-CAH than women admitted to CAH (3.400, 95% CI: 3.3828-3.4172, p < 0.0001). A statistically significant difference in mortality was observed in men, albeit smaller (Figure 2). The AAMR for hospitalized HF men with access to CAH was 49.4 (95% CI: 41.8-56.9), compared to 51.6 (95% CI: 43.6-59.7) for men without CAH in their counties.

**Figure 3:**
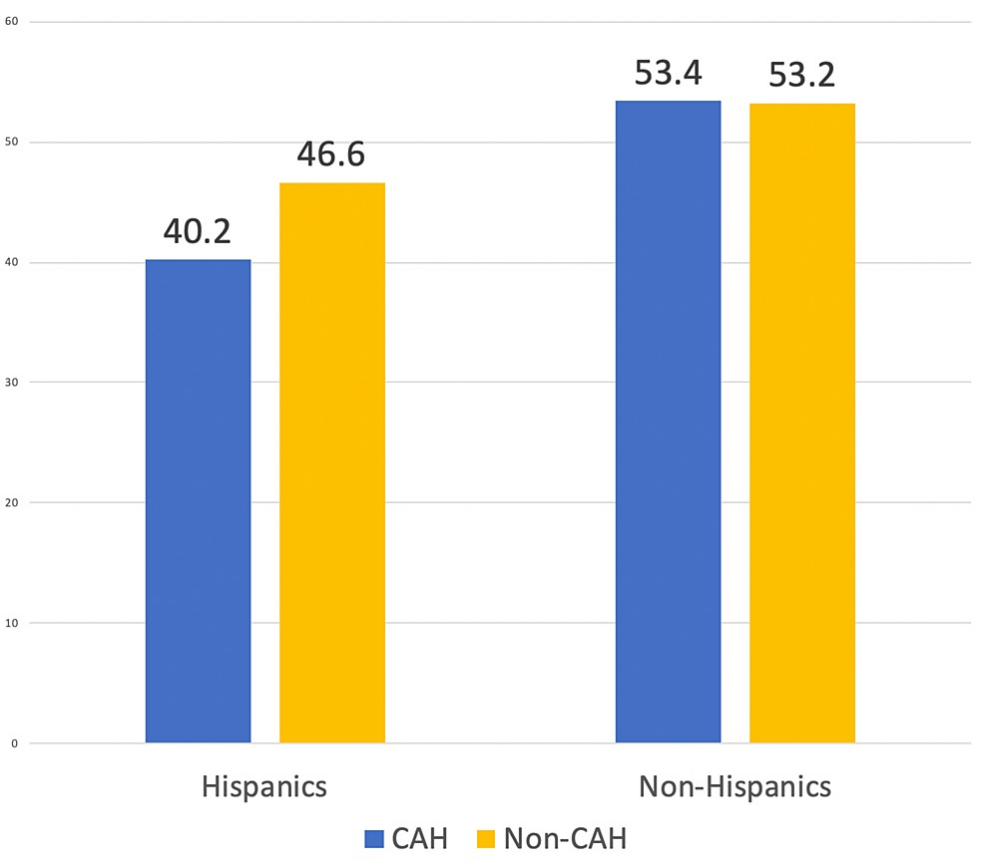
The bar graph shows a significant difference in mortality rate within the Hispanic population of 6.400 (95% CI: 6.3770 to 6.4230, p < 0.0001). In the other ethnic group, a statistically significant difference was observed favoring non-CAH: -0.200 (95% CI: -0.2149 to 0.1851, p < 0.0001). CAH, Critical Access Hospital

When examining the data by race or ethnicity, the most significant difference in mortality rate was observed within the Hispanic population (Figure 3). The AAMR for Hispanics in the study group was 40.2 (95% CI: 32.8-47.6) and 46.6 (95% CI: 36.6-58.4) in the control group. The two groups had a statistically significant difference of 6.400 (95% CI: 6.3770-6.4230, p < 0.0001). In an analysis of different ethnicities, including White, Black, Asian, and American Indian, there was a small but statistically significant difference between the control and study groups (Table 1). However, this difference in AAMR was reversed compared to the observed difference in Hispanics. AAMR was higher in counties with the availability of CAH (53.4; 95% CI: 46.9 to 59.9) compared to the control group (53.2; 95% CI: 46.8 to 59.5), with a difference in the mortality rate of -0.200 (95% CI: -0.2149 to 0.1851, p < 0.0001).

**Table 1:** Mortality difference in HF patients between CAH and non-CAH when adjusted to different covariants CAH, Critical Access Hospital; HF, heart failure; NA, not applicable

		CAH	Number of deaths	Population	Crude rate	Age-adjusted rate with CI	Difference observed based on critical access	p-value
Male		Yes	169	396,142	42.7	49.4 (41.8-56.9)	2.2000	p < 0.001
		No	160	333,291	48	51.6 (43.6-59.7)		
Female		Yes	209	338,092	61.8	47.4 (40.9-53.8)	3.400	p < 0.001
		No	197	280,476	70.2	50.8 (43.6-57.9)		
Hispanic		Yes	116	369,710	31.4	40.2 (32.8-47.6)	6.400	p < 0.001
		No	79	243,855	32.4	46.6 (36.6-58.4)		
White		Yes	368	692,474	53.1	49.4 (44.3-54.4)	3.000	p < 0.001
		No	345	567,508	60.8	52.4 (46.9-58.0)		
Black		Yes	10	26,181	38.2	NA	1.300	p < 0.001
		No	12	30,377	39.5	NA		
Age	55-64	Yes	26	128,998	20.2	NA	10.2	p < 0.001
		No	33	108,484	30.4	NA		
	65-74	Yes	47	88,225	53.3	NA	11.5	p < 0.001
		No	48	74,130	64.8	NA		
	75-84	Yes	111	50,680	219	NA	-20.6	p < 0.001
		No	89	44,863	198.4	NA		
	≥85	Yes	184	18,475	995.9	NA	-17.9	p < 0.001
		No	165	16,871	978	NA		
Overall		Yes	378	734,234	51.5	48.7 (43.8-53.6)	3.200	p < 0.001
		No	357	613,767	58.2	51.9 (46.5-57.3)		

For White individuals, a subgroup analysis was performed. Those with access to neighboring CAH had an AAMR of 49.4 per 100,000 individuals (95% CI: 44.3-54.4, SE: 2.6), while those without CAH had an AAMR of 52.4 (95% CI: 46.9-58.0, SE: 2.8), resulting in a statistically significant difference in the mortality rate of 3.000 per 100,000 persons (95% CI: 2.9905-3.0094, p < 0.0001). Due to limited mortality and data, only CMRs could be calculated in Black individuals. A statistically significant CMR difference was found between both groups (38.2 in the CAH group and 39.5 in the non-CAH group, difference: 1.300, p < 0.0001). Subgroup analysis could not be performed for underrepresented groups in this population, such as Asian and American-Indian, due to limited data.

**Figure 4:**
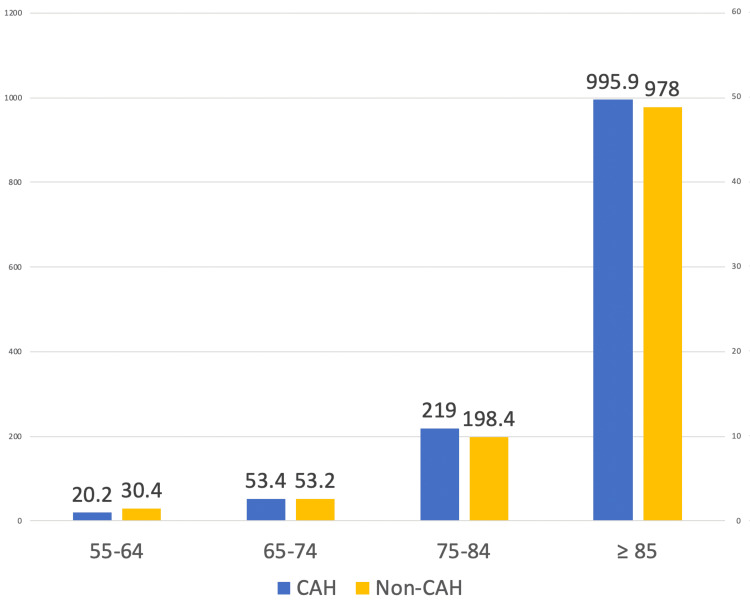
The bar graph shows that in the ≥85 age group and 75-84 age group, the CMR was higher in CAH compared to non-CAH (-17.90, 95% CI: -19.4592 to -16.3408, p < 0.0001 and -20.600 (95% CI: -20.8655 to -20.3345), respectively. This pattern is reversed in the younger population groups, with CMR higher in non-CAH compared to CAH (10.200 (95% CI: 10.1625-10.2375, p < 0.001) and 11.500 (95% CI: 11.4168-11.5832, p < 0.001)), in the 55-64 and 65-74 age groups, respectively. CAH, Critical Access Hospital; CMR, crude mortality rate

When comparing the mortality rates of different age groups in 10-year intervals ranging from 25 to 85 years old and above, we found no reported deaths in patients younger than 55 years, and no data was available on patients ≤45 years old. An interesting finding was that in the ≥85 age group and 75-84 age group, the CMR was higher in CAH compared to non-CAH (-17.90, 95% CI: -19.4592 to -16.3408, p < 0.0001 and -20.600 (95% CI: -20.8655 to -20.3345), respectively (Figure 4). This pattern is reversed in the younger population groups, with CMR higher in non-CAH compared to CAH (10.200 (95% CI: 10.1625-10.2375, p < 0.001) and 11.500 (95% CI: 11.4168-11.5832, p < 0.001) in the 55-64 and 65-74 age groups, respectively. Table 1 summarizes the results obtained in our study.

## Discussion

The CAH program was established by the Balanced Budget Act of 1997 to increase access to healthcare in rural communities and keep essential services available to the local rural population [7]. The ultimate focus of this program was to promote well-being and decrease morbidity and mortality in the community that the CAHs serve. Despite that, multiple publications and analyses indicated that the quality of critical care provided to the rural population needed substantial improvement [3,4,8].

Initial works by Joynt et al. demonstrated excess all-cause mortality of 1.4% in rural hospitals using data from the 1980s, comparable with differences noted by the Medicare Payment Advisory Committee using data from 2003 [3]. In a more recent retrospective analysis, the same team of researchers showed that the mortality rate in patients with acute myocardial infarction, HF, and pneumonia discharged from CAH between 2008 and 2009 was higher compared to non-CAH [4]. The authors included lack of adequate personnel, inadequate outpatient care, lack of access to ongoing primary care, post-hospitalization follow-up, rehabilitation, and home-based care, to name a few, as possible reasons for their results. On the other hand, when rurality was added to the models and rural CAHs were compared to rural non-CAHs, the excess mortality at CAHs decreased.

Our study aimed to focus primarily on the rural setting of West Texas to prove that CAH designation in underserved rural communities delivered on the purpose it intended to serve. Furthermore, we planned to focus on the HF population, bearing in mind the extreme burden of this disease nationwide and worldwide.

The American Heart Association estimated that there were 6.2 million people with HF in the United States between 2013 and 2016 [9]. The prevalence of HF in the United States is projected to rise over the next four decades, with an estimated 772,000 new HF cases projected in 2040 [10] and 8 million prevalent cases by 2030 [11]. Additionally, HF mortality remains high despite advances in diagnosis and treatment [12]. Furthermore, hospitalization is an important marker of poor prognosis. In the Candesartan in Heart Failure-Assessment of Reduction in Mortality and Morbidity (CHARM) study, the mortality rate increased after HF hospitalizations, even after adjustment for baseline predictors of death [13].

Keeping all the aforementioned in mind, we aimed to ascertain if CAH designation can improve mortality rates in the HF population of rural West Texas. Of West Texas’s 25 rural county hospitals, 13 had critical care access, whereas 12 did not. By analyzing the data from 2010 to 2020, we concluded that the AAMR in CAH was significantly lower than that in non-CAH. When adjusted to sex and race, the results consistently showed significantly lower AAMR in CAH. The data clearly showed that West Texas rural county hospitals that received CAH designation performed better in terms of mortality rates in the HF population compared to non-CAH.

While further investigating the topic and researching the factors that contributed to positive results in our study, we concluded that significant efforts had been made in Texas overall and in West Texas, in particular, to overcome challenges and improve healthcare delivery in rural areas, including workforce shortages, difficulty in recruiting professional personnel such as nurses, physicians, and specialists to underserved areas [14], inadequate outpatient care, aging demographics, and other obstacles [15-17].

One of the key strategies employed by rural West Texas hospitals is the integration of electronic health record (EHR) systems. By implementing EHR systems, rural hospitals improved patient care coordination, streamlined data management, and enhanced communication among healthcare providers. This shift to digital records enabled more accessible access to patient information and facilitated post-hospitalization follow-ups, rehabilitation, and home-based care.

In addition to EHR systems, rural hospitals in Texas are embracing telemedicine capabilities and high-speed internet connectivity. Through initiatives like the Texas Rural Broadband Expansion Program, broadband infrastructure and services are being expanded in underserved rural communities. This improved connectivity enhanced the availability and usage of EHR systems in rural hospitals. It also enabled the adoption of telehealth technology, allowing rural populations to access remote consultation, monitoring, and specialty care without traveling long distances [18].

Furthermore, programs like the Rural Health Information Technology (HIT) Assistance Program and Meaningful Use and Incentive Programs have assisted rural healthcare providers, including hospitals, in adopting and effectively utilizing health information technology. These initiatives provided technical support, training, and guidance on implementing and optimizing EHR systems [19,20].

Partnerships between CAHs and larger hospitals, their incorporation into healthcare systems, and joint ventures have also contributed to developing and providing services that CAHs may not be able to offer independently [21].

However, as mentioned earlier, challenges persist. Rural areas in Texas continue to struggle with recruiting and retaining healthcare professionals due to lower population density, limited resources, and fewer professional opportunities [22]. Additionally, rural communities face higher rates of poverty, limited access to education, and a higher prevalence of chronic diseases compared to urban areas, contributing to health disparities [23].

Measures to devote to quality improvement need to be more actively incorporated. Reporting Health Quality Alliance (HQA) data by CAH could have provided an understanding of the specific indicators associated with better outcomes in the HF population admitted to CAH in the West Texas region, but the fact that CAHs are not required to report HQA data understates the actual differences in care and hinders adequate quality analysis [24].

Ongoing policy and funding challenges, emerging violence in rural communities, cyber threats, and the opioid epidemic are significant issues that the rural healthcare system and CAHs must address [25].

Despite these challenges, the integration of technology, expansion of broadband access, and collaborative efforts are gradually improving the healthcare landscape in rural Texas. Continued investments in healthcare infrastructure, workforce development, and addressing social determinants of health are crucial to ensuring equitable access to quality care in rural communities.

Last but not least, our study has certain limitations. One notable limitation of this study is the reliance on administrative data and ICD-10 codes to identify HF patients. The study lacks detailed clinical information, such as specific comorbidities, severity of HF, or adherence to treatment protocols. This absence of granular clinical data may restrict the depth of analysis and limit the ability to account for certain confounding factors that could influence mortality rates. Future research incorporating more comprehensive clinical data would provide a more nuanced understanding of the relationship between CAHs and HF outcomes in rural West Texas.

## Conclusions

CAHs are vital in ensuring access to medical services for the most vulnerable rural populations. Further investigation is needed to ascertain which of the 12 non-CAHs in West Texas might be eligible to receive a critical access designation and the limitations of providing essential care services in these hospitals. Additionally, our study mainly focused on the HF population, and outcome analysis of other acute conditions can further alleviate the significance of this topic in the future. Our data makes a solid argument for providing additional resources to the most vulnerable patients living in the West Texas rural area.
